# Association of rs780094 in *GCKR* with Metabolic Traits and Incident Diabetes and Cardiovascular Disease: The ARIC Study

**DOI:** 10.1371/journal.pone.0011690

**Published:** 2010-07-22

**Authors:** Mark Bi, Wen Hong Linda Kao, Eric Boerwinkle, Ron C. Hoogeveen, Laura J. Rasmussen-Torvik, Brad C. Astor, Kari E. North, Josef Coresh, Anna Köttgen

**Affiliations:** 1 School of Biological Sciences, University of Nebraska at Lincoln, Lincoln, Nebraska, United States of America; 2 Department of Epidemiology, Johns Hopkins Bloomberg School of Public Health, Baltimore, Maryland, United States of America; 3 Human Genetics Center and Division of Epidemiology, University of Texas Health Science Center at Houston, Houston, Texas, United States of America; 4 Department of Medicine, Baylor College of Medicine, Houston, Texas, United States of America; 5 Division of Epidemiology and Community Health, University of Minnesota, Minneapolis, Minnesota, United States of America; 6 Department of Epidemiology and Carolina Center for Genome Sciences, University of North Carolina, Chapel Hill, North Carolina, United States of America; 7 Renal Division, University Hospital Freiburg, Freiburg, Germany; Innsbruck Medical University, Austria

## Abstract

**Objective:**

The minor *T*-allele of rs780094 in the glucokinase regulator gene (*GCKR*) associates with a number of metabolic traits including higher triglyceride levels and improved glycemic regulation in study populations of mostly European ancestry. Using data from the Atherosclerosis Risk in Communities (ARIC) Study, we sought to replicate these findings, examine them in a large population-based sample of African American study participants, and to investigate independent associations with other metabolic traits in order to determine if variation in *GKCR* contributes to their observed clustering. In addition, we examined the association of rs780094 with incident diabetes, coronary heart disease (CHD), and stroke over up mean follow-up times of 8, 15, and 15 years, respectively.

**Research Design and Methods:**

Race-stratified analyses were conducted among 10,929 white and 3,960 black participants aged 45–64 at baseline assuming an additive genetic model and using linear and logistic regression and Cox proportional hazards models.

**Results:**

Previous findings replicated among white participants in multivariable adjusted models: the *T*-allele of rs780094 was associated with lower fasting glucose (p = 10^−7^) and insulin levels (p = 10^−6^), lower insulin resistance (HOMA-IR, p = 10^−9^), less prevalent diabetes (p = 10^−6^), and higher CRP (p = 10^−8^), 2-h postprandial glucose (OGTT, p = 10^−6^), and triglyceride levels (p = 10^−31^). Moreover, the *T*-allele was independently associated with higher HDL cholesterol levels (p = 0.022), metabolic syndrome prevalence (p = 0.043), and lower beta-cell function measured as HOMA-B (p = 0.011). Among black participants, the *T*-allele was associated only with higher triglyceride levels (p = 0.004) and lower insulin levels (p = 0.002) and HOMA-IR (p = 0.013). Prospectively, the *T*-allele was associated with reduced incidence of diabetes (p = 10^−4^) among white participants, but not with incidence of CHD or stroke.

**Conclusions:**

Our findings indicate rs780094 has independent associations with multiple metabolic traits as well as incident diabetes, but not incident CHD or stroke. The magnitude of association between the SNP and most traits was of lower magnitude among African American compared to white participants.

## Introduction

Metabolic traits that are risk factors for cardiovascular disease (CVD) commonly occur together [1] and in a specific combination are known as metabolic syndrome. Metabolic syndrome has been linked to increased all-cause mortality, CVD, and diabetes mellitus (DM)[1]–[4]. While the individual metabolic traits have heritable components, it is less clear whether such clustering has a common biologic basis [4], [5]. Genome-wide association studies (GWAS) have discovered susceptibility loci for some of the individual components such as glucose and lipid levels [6]–[8] (and http://www.genome.gov/GWAstudies/). Genetic loci associated with serum levels of biomarkers related to the metabolic syndrome such as C-reactive protein (CRP) have also been identified[9]. In light of the clustering of metabolic risk factors, however, it is of particular interest to investigate genetic loci that have demonstrated association with multiple metabolic traits.

The glucokinase regulator gene (*GCKR*) encodes for the glucokinase regulator protein (GKRP), which regulates glycolysis primarily in liver hepatocytes but is also found in small quantities in pancreatic beta cells [10], [11]. Common variants in *GCKR*, most often the minor *T*-allele of the common single nucleotide polymorphism (SNP) rs780094, have been reported by multiple candidate gene and GWA studies to be associated with decreased fasting glucose[12]–[19] and insulin levels [12], [16], [18], lower insulin resistance[12], [15]–[18], [20], and decreased prevalence of type 2 diabetes [12], [15], [16], [18]. At the same time, the *T* allele has also been found to be associated with increased serum triglyceride[5], [12], [15]–[18], [21]–[29], CRP[9], [17], [29], and 2 hour OGTT glucose levels[12], [30]. Some studies have also found evidence of an association with beta cell function as measured by HOMA-B [12], [25] and HDL cholesterol concentration[17], [26], although these associations have not been observed in all studies [13], [15], [18], [21], [23], [24], [27], [29]. Despite its association with multiple metabolic risk factors, studies have observed no association with cardiovascular disease[17], [21], [27], [31], [32].

To date, most of these studies have been conducted in populations of European[5], [9], [12], [15], [16], [18]–[21], [23], [24], [26], [27], [29]–[33] and Asian ancestry[13], [14], [25], [34]. However, it is of interest to study metabolic risk factors in populations with particularly high prevalence of diabetes and the metabolic syndrome, including Hispanics, Native Americans, and African Americans. Only one study thus far has included a population of African ancestry, where an association of rs780094 with plasma triglyceride levels but not fasting glucose levels was observed[17].

We therefore studied the association of rs780094 with metabolic traits in 10,929 self-reported white and 3,960 black participants of the Atherosclerosis Risk in Communities (ARIC) Study with the following objectives: (1) to replicate the previously observed associations in white participants in a large population-based study sample; (2) to explore these associations in black study participants; (3) to characterize the association of rs780094 with additional metabolic traits, including waist circumference, systolic blood pressure, HDL cholesterol concentration, homeostasis model assessment of beta-cell function (HOMA-B), and metabolic syndrome prevalence; and (4) to examine its prospective association with incident diabetes, coronary heart disease (CHD), and stroke.

## Methods

### Study Population

The ARIC Study is a prospective, population-based study investigating the etiology and epidemiology of atherosclerosis. From 1987 to 1989, 15,792 adults aged 45–64 years old were recruited by probability sampling from 4 US locations. All participants gave written informed consent, and the study was approved by the local Research Ethics Committees of the four ARIC study centers (University of Minnesota, Minneapolis; University of Mississippi, Jackson; University of North Carolina, Chapel Hill; Johns Hopkins University, Baltimore). Participants underwent interviews and examinations at baseline and roughly every 3 years afterwards for a total of 4 study visits. Annual follow-up using telephone surveys and information collected from death certificates and hospital discharge records is ongoing. A complete description of the ARIC Study is available elsewhere [35].

For the current study, participants were excluded for the following reasons: did not self-identify as white or black (n = 48), no genotyping consent (n = 45), or missing genotype (n = 810). After exclusions, a total of 3,960 black and 10,929 white participants remained and were included in univariate analyses. Multivariate analyses were conducted separately among 10,601 white and 3,513 black participants with complete outcome and covariate information. Subsets of both samples were used in analyses of outcomes only measured at study visit 4 (n = 7,703 [2h-OGTT] and n = 8,131 [CRP] for white participants; n = 1,450 [2h-OGTT] and n = 2,123 [CRP] for black participants).

### Genotyping

rs780094 was genotyped at the central ARIC DNA laboratory using the TaqMan Assay-by-Design system (Applied Biosystems; Foster City, CA). Call rate was 93.3% in black participants and 95.5% in white participants. Genotype distributions conformed to expectations under Hardy-Weinberg Equilibrium (p-exact >0.2 for each race). Genotyping in a set of 750 blind duplicates showed good reproducibility (kappa coefficient 0.98).

### Study Outcomes

Primary cross-sectional outcomes were traits previously observed to be associated with rs780094, including serum fasting glucose, fasting insulin, HOMA-IR, triglycerides, DM, and CRP. We further examined additional Adult Treatment Panel III (ATP III) criteria of the metabolic syndrome (systolic blood pressure, HDL cholesterol, and waist circumference)[2]. Secondary cross-sectional outcomes were postprandial glucose measured by an oral-glucose tolerance test (OGTT), metabolic syndrome, and HOMA-B. Baseline measurements were used for all outcomes except those only available from the 4^th^ visit (CRP and post-OGTT glucose). Diabetes was defined as a fasting serum glucose concentration of ≥126 mg/dl after ≥8 hours of fasting, ≥200 mg/dl after <8 hours of fasting, self-reported physician diagnosis, or current intake of diabetes medication. Metabolic syndrome was defined as fulfillment of three or more ATP III criteria for the metabolic syndrome (waist circumference ≥102 cm (men) or ≥88 cm (women), triglyceride levels ≥150 mg/dl, HDL cholesterol <40 (men) or <50 mg/dl (women), blood pressure ≥130/85 mm Hg, fasting glucose ≥100 mg/dl).

The primary prospective outcome was incident DM[36]. Secondary prospective outcomes were incident stroke and incident CHD defined as myocardial infarction (MI), fatal CHD, silent MI from EKG, or cardiac revascularization procedure[37], [38].

### Demographic and Clinical Measurements

Race, age, sex and smoking status were self-reported at study visits. Current medication usage was self-reported and validated by inspection of containers. Blood pressure was measured using a random zero sphygmomanometer and analyzed as the average of the 2^nd^ and 3^rd^ reading. Blood samples were drawn, frozen at −70°C, and shipped to ARIC laboratories for storage and testing. Fasting serum glucose levels were measured using the hexokinase–glucose-6-phosphate dehydrogenase method, plasma triglycerides by an enzymatic method, plasma HDL cholesterol by dextran-magnesium precipitation and serum uric acid by uricase oxidation[39]. Serum insulin was determined using radioimmunoassay at baseline and an enzyme-linked immunosorbent assay (Boehringer Mannheim Corporation, Mannheim, Germany) at visit 4.

At visit 4 only, a standard 75 g OGTT was administered to participants not taking diabetes medication. Serum high-sensitivity C-reactive protein (hsCRP) was determined using an immunoturbidimetric assay on a Siemens BNII analyzer (Dade Behring, Deerfield, Ill).

A complete description of ARIC protocols is available from the ARIC operations manual[39].

### Statistical Analyses

Race-stratified analyses were conducted assuming an additive genetic model based on previous results[16], [18]. Cross-sectional outcomes were tested for association with rs780094 using simple and multivariable-adjusted linear and logistic regression as applicable. Incident outcomes were tested using multivariable-adjusted Cox proportional hazards models.

Univariate regression models were first used to replicate previously described associations. Two multivariate models were explored: (1) adjusted for age, sex, and study center; (2) further adjusted for fasting glucose, insulin, and triglyceride levels. Fasting glucose, insulin, and triglyceride levels were selected on the basis of their consistently replicated association with rs780094. A trait was excluded as a covariate when being evaluated as an outcome. Covariate measurements were always used from the same visit as measurements of the tested outcome. Because DM was diagnosed based on glucose levels, and HOMA-IR and HOMA-B are functions of fasting glucose and insulin, these traits were not adjusted for each other. As fasting glucose and triglyceride levels are used to define the presence of the metabolic syndrome, associations with metabolic syndrome were not adjusted for these two parameters.

Sensitivity analyses were conducted using a sub-sample of participants who did not have diabetes and reported taking no lipid-lowering medications at baseline. Additional sensitivity analyses were also conducted using the means of repeated measurements from different study visits.

Prospective analyses were conducted accordingly; baseline measurements were used for covariates.

Since this rs780094 is an extensively studied candidate SNP, a threshold of p = 0.05 was used to indicate statistical significance.

Additionally, as the activity of the glucokinase regulator protein is enhanced by fructose-6-phosphate but inhibited by fructose-1-phosphate[10], we tested for an interaction between rs780094 and total fructose intake in associations with glucose, triglyceride, and insulin levels.

All analyses were performed using Stata version 10.0 (StataCorp, College Station, TX).

## Results

The frequency of the rs780094 *T*-allele was 39.7% among white participants and 17.8% among black participants. Genotype and allele distributions are shown in Table 1.

**Table 1:** Distribution of rs780094 in 3,960 Black and 10,929 White Atherosclerosis Risk in Communities Study Participants.

	Black	White
**Genotypes**		
* CC*	67.9 (2,690)	36.3 (3,970)
* CT*	28.6 (1,134)	48.0 (5,245)
* TT*	3.4 (136)	15.7 (1,714)
**Alleles**		
* C*	82.2	60.3
* T*	17.8	39.7

*Note:* Values expressed as percent (count). Exact p-values for Hardy-Weinberg Equilibrium were 0.22 for white and 0.78 for black participants.

Study sample characteristics of white participants by rs780094 genotype are presented in Table 2. Previously observed associations of rs780094 and metabolic traits reached statistical significance among white participants in univariate analyses: each additional copy of the *T*-allele was associated with higher triglyceride (p = 5×10^−22^), CRP (p = 3×10^−8^), and post-OGTT glucose levels (p = 0.009), higher prevalence of the metabolic syndrome (p = 0.016), and with lower fasting glucose (p = 0.002), fasting insulin (p = 0.007) and HOMA-IR (p = 0.006). There was a trend towards lower DM prevalence (p = 0.057). Demographic characteristics, including age, sex, drinking and smoking status, and BMI did not differ with respect to genotype.

**Table 2:** Distribution of Study Traits in 10,929 White Atherosclerosis Risk in Communities Study Participants by rs780094 Genotype.

	n	*CC*	*CT*	*TT*	P-trend
Male (%)	10,929	46.9	46.9	47.8	0.586
Age (yr)	10,929	54.3	54.3	54.5	0.244
BMI (kg/m^2^)	10,922	27.0±5.0	27.0±4.8	27.0±4.9	0.788
Current smoker (%)	10,922	24.2	25.4	24.5	0.564
Current drinker (%)	10,913	64.8	64.4	64.3	0.677
Fasting glucose (mg/dl)	10,683	105.3±29.9	105.2±29.0	102.2±25.9	0.002
Diabetes mellitus (%)	10,911	9.1	9.8	6.8	0.057
2 hr post-OGTT glucose (mg/dl)	7,129	137.1±54.3	137.9±52.8	142.5±51.9	0.009
Insulin (pmol/l)	10,924	88.9±155	88.3±161	75.3±70	0.007
HOMA-IR	10,683	3.46±5.2	3.47±6.3	2.92±4.0	0.006
HOMA-B	10,683	113.8±372	105.3±86	103.7±70	0.077
Triglycerides (mmol/l)	10,914	1.44±0.9	1.59±1.1	1.70±1.2	5.2×10^−22^
HDL cholesterol (mg/dl)	10,914	51.0±16.9	50.1±16.8	50.6±16.6	0.129
Systolic blood pressure (mmHg)	10,924	118.3±16.9	118.3±16.8	119.4±17.7	0.071
Waist circumference (cm)	10,919	96.1±13.7	96.3±13.2	96.4±13.3	0.431
C-reactive protein (mg/l)	8,370	3.63±5.0	4.27±6.6	4.65±7.3	3.1×10^−8^
Metabolic syndrome (%)	10,878	36.4	39.0	39.2	0.016

*Note:* Data are mean±SD for quantitative variables and percentages for categorical variables. Sample sizes are the number of participants with non-missing information for the respective variable out of 10,929 genotyped individuals (3,970 *CC*, 5,245 *CT*, 1,714 *TT*). All measurements are from baseline with the exceptions of 2 hr post-OGTT glucose and C-reactive protein, which were only measured at the 4^th^ visit. P values given for association with rs780094 in linear regression for quantitative traits and logistic regression for qualitative traits.

Results of multivariable adjusted analyses in white participants are shown in Table 3. Adjusting for age, sex, and study center (model 1), all previously reported associations (fasting glucose, fasting insulin, HOMA-IR, post-OGTT glucose, triglycerides, DM, and CRP) were confirmed. A trend towards an association of the *T* allele with lower HOMA-B (p = 0.063) was also observed; the association with metabolic syndrome was nominally significant (p = 0.043). Additionally, when the study sample was dichotomized by fructose intake at the median, the association of rs780094 with triglyceride, glucose, and insulin levels appeared to be of greater magnitude per *T* allele among individuals with higher fructose intake. However, the differences were not statistically significant (all p-interaction >0.13).

**Table 3:** Adjusted Associations between rs780094 and Study Outcomes in White Atherosclerosis Risk in Communities Study Participants.

	Model 1*		Model 2*	
**Quantitative traits**	**Effect/** ***T*** ** allele ± SE**	**P**	**Effect/** ***T*** ** allele ± SE**	**P**
Triglycerides (mmol/l)	+0.13±0.01	2.9×10^−21^	+0.16±0.01	2.4×10^−31^
Fasting glucose (mg/dl)	−1.39±0.4	0.001	−1.93±0.4	2.3×10^−7^
Fasting insulin (pmol/l)	−5.07±1.4	2.9×10^−4^	−6.29±1.3	1.9×10^−6^
HOMA-IR	−0.24±0.1	0.002	−0.45±0.1	2.2×10^−9^
HOMA-B	−6.14±3.3	0.063	−8.36±3.3	0.011
Waist circumference (cm)	+0.06±0.2	0.747	+0.01±0.2	0.940
HDL cholesterol (mg/dl)	−0.21±0.2	0.317	+0.43±0.2	0.022
Systolic blood pressure (mmHg)	+0.39±0.2	0.090	+0.30±0.2	0.188
C-reactive protein (mg/l)	+0.53±0.1	7.3×10^−8^	+0.56±0.1	1.6×10^−8^
2 hr post-OGTT glucose (mg/dl)	+1.99±0.9	0.025	+3.06±0.7	2.8×10^−6^
**Categorical traits**	**OR/** ***T*** ** allele (95% CI)**	**P**	**OR/** ***T*** ** allele (95% CI)**	**P**
Diabetes mellitus	0.89 (0.80–0.98)	0.019	0.78 (0.71–0.87)	5.2×10^−6^
Metabolic syndrome	1.06 (1.00–1.12)	0.043	1.17 (1.10–1.25)	1.5×10^−6^
**Incident events**	**HR/** ***T*** ** allele (95% CI)**	**P**	**HR/** ***T*** ** allele (95% CI)**	**P**
Diabetes mellitus	0.89 (0.81–0.98)	0.014	0.85 (0.77–0.93)	4.6×10^−4^
Coronary heart disease	0.97 (0.91–1.04)	0.422	0.98 (0.91–1.05)	0.523
Stroke	0.94 (0.82–1.08)	0.363	0.96 (0.83–1.11)	0.570

*Note: Model 1 adjusted for age, sex, study center; model 2 adjusted for age, sex, study center, fasting glucose, fasting insulin, triglycerides. Metabolic syndrome was not adjusted for glucose and triglycerides in model 2. HOMA-IR, HOMA-B, and DM not adjusted for fasting glucose and insulin in model 2. All cross-sectional associations evaluated using baseline measurements (n = 10,601), except for 2 hr post-OGTT glucose (n = 7,073) and C-reactive protein (n = 8,131), which were only measured at visit 4. 935 participants out of 9,230 developed DM over mean follow-up of 8 years; 1,705 out of 10,600 developed CHD and 418 out of 10,600 experienced a stroke over mean follow-up of 15 years.

To evaluate the individual contribution of these traits, we included fasting glucose, insulin, and triglyceride levels as additional covariates (model 2, Table 3). Most significant associations were strengthened upon adjustment. Associations of the *T* allele with lower HOMA-B (p = 0.011) and higher HDL cholesterol (p = 0.022) were also observed. The T allele showed significant association with higher prevalence of the metabolic syndrome (p = 10^−6^), which disappeared when adjustment for triglycerides was performed. No relationships with systolic blood pressure and waist circumference were observed under either of the models.

Characteristics of black ARIC participants by genotype are listed in Table 4. The *T*-allele of rs780094 was nominally associated only with higher triglyceride levels (p = 0.05) and lower HOMA-IR (p = 0.023) and metabolic syndrome prevalence (p = 0.037) in univariate models. In multivariable adjusted analyses, the *T*-allele additionally showed association with lower insulin levels (p = 0 .002); the relationship with metabolic syndrome became insignificant (Table 5).

**Table 4:** Distribution of Study Traits in 3,960 Black Atherosclerosis Risk in Communities Study Participants.

	n	*CC*	*CT*	*TT*	P
Male (%)	3,960	38.8	37.2	36.0	0.293
Age (yr)	3,960	53.5	53.5	52.9	0.574
BMI (kg/m^2^)	3,951	29.7±6.2	29.4±6.1	29.2±6.6	0.058
Current smoker (%)	3,952	30.1	28.7	36.0	0.807
Current drinker (%)	3,922	32.4	32.6	30.8	0.788
Fasting glucose (mg/dl)	3,590	113.3±45.3	110.6±45.1	115.7±52.3	0.390
Diabetes mellitus (%)	3,878	20.1	17.7	22.6	0.367
2 hr post-OGTT glucose (mg/dl)	1,465	141.5±59.3	144.1±59.9	150.6±73.8	0.255
Insulin (pmol/l)	3,875	150.5±299	127.5±254	183.2±697	0.381
HOMA-IR	3,590	5.74±16.1	4.59±7.1	4.46±6.1	0.023
HOMA-B	3,590	132.2±783	143.2±344	117.7±75	0.845
Triglycerides (mmol/l)	3,819	1.28±0.9	1.31±1.0	1.44±1.3	0.050
HDL cholesterol (mg/dl)	3,818	54.6±17.5	55.5±17.4	55.6±18.0	0.120
Systolic blood pressure (mmHg)	3,957	128.7±21.2	128.2±21.6	129.3±20.9	0.705
Waist circumference (cm)	3,953	99.4±15.3	98.7±14.7	98.6±16.0	0.156
C-reactive protein (mg/l)	2,374	5.91±8.3	5.99±7.7	6.29±9.2	0.695
Metabolic syndrome (%)	3,786	44.3	40.3	40.9	0.037

*Note:* Data are mean ± SD for quantitative variables and percentages for categorical variables. Sample sizes are the number of participants with non-missing information for the respective variable out of 3,960 genotyped individuals (2,690 *CC*, 1,134 *CT*, 136 *TT*). All measurements are from baseline with the exceptions of 2 hr post-OGTT glucose and C-reactive protein, which were only measured at the 4^th^ visit. P values given for association with rs780094 in linear regression for quantitative traits and logistic regression for qualitative traits.

**Table 5:** Adjusted Associations between rs780094 and Study Outcomes in Black Atherosclerosis Risk in Communities Study Participants.

	Model 1*		Model 2*	
**Quantitative traits**	**Effect/** ***T*** ** allele ± SE**	**P**	**Effect/** ***T*** ** allele ± SE**	**P**
Triglycerides (mmol/l)	+0.06±0.03	0.016	+0.07±0.02	0.004
Fasting glucose (mg/dl)	−0.86±1.4	0.537	−0.68±1.3	0.609
Fasting insulin (pmol/l)	−16.18±5.6	0.004	−16.53±5.4	0.002
HOMA-IR	−0.93±0.4	0.030	−1.06±0.4	0.013
HOMA-B	+4.99±20.9	0.811	+3.43±20.9	0.870
Waist circumference (cm)	−0.60±0.5	0.192	−0.46±0.5	0.301
HDL cholesterol (mg/dl)	+0.56±0.5	0.284	+0.81±0.5	0.100
Systolic blood pressure (mmHg)	−0.35±0.6	0.584	−0.34±0.6	0.587
C-reactive protein (mg/l)	+0.30±0.3	0.353	+0.32±0.3	0.314
2 hr post-OGTT glucose (mg/dl)	+3.19±2.9	0.277	+1.94±1.8	0.279
**Categorical traits**	**OR/** ***T*** ** allele (95% CI)**	**P**	**OR/** ***T*** ** allele (95% CI)**	**P**
Diabetes mellitus	0.98 (0.83–1.16)	0.843	0.95 (0.80–1.13)	0.569
Metabolic syndrome	0.89 (0.79–1.01)	0.071	0.96 (0.83–1.10)	0.536
**Incident events**	**HR/** ***T*** ** allele (95% CI)**	**P**	**HR/** ***T*** ** allele (95% CI)**	**P**
Diabetes mellitus	0.91 (0.77–1.08)	0.284	0.94 (0.79–1.12)	0.494
Coronary heart disease	1.10 (0.93–1.31)	0.279	1.08 (0.91–1.29)	0.368
Stroke	0.97 (0.78–1.21)	0.791	0.96 (0.77–1.20)	0.750

*Note: Model 1 adjusted for age, sex, study center; model 2 adjusted for age, sex, study center, fasting glucose, fasting insulin, triglycerides. Metabolic syndrome was not adjusted for glucose and triglycerides in model 2. HOMA-IR, HOMA-B, and DM not adjusted for fasting glucose and insulin in model 2. All cross-sectional associations evaluated using baseline measurements (n = 3,513, except for 2 hr post-OGTT glucose (n = 1,450) and C-reactive protein (n = 2,123), which were only measured at visit 4. 478 participants out of 2,626 developed DM over mean follow-up of 8 years; 408 out of 3,513 developed CHD and 273 out of 3,513 experienced a stroke over mean follow-up of 15 years.

**Figure 1:**
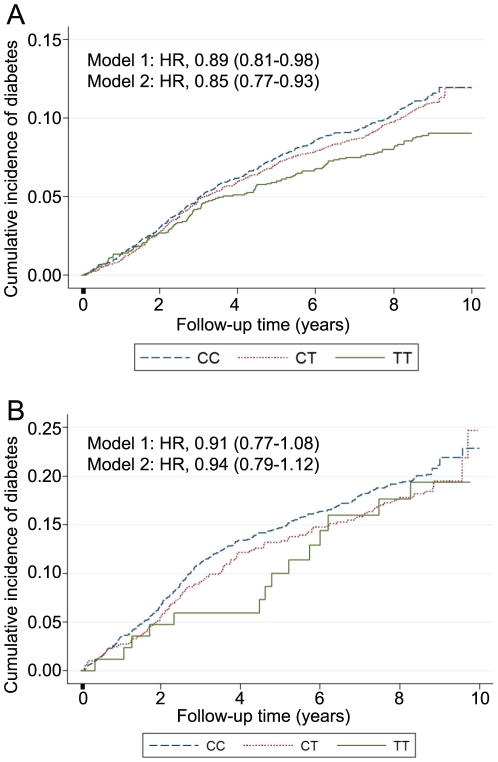
Cumulative incidence of diabetes stratified by genotype among 9,356 white (panel A) and 2,626 black (panel B) Atherosclerosis Risk in Communities participants over a mean of 8 years of follow-up.

Over a mean follow-up period of 8 years, 935 out of 9,230 white participants without diabetes at baseline developed diabetes. Figure 1A shows the cumulative incidence of diabetes stratified by rs780094 genotype. In Cox proportional hazards models, relative hazards were significantly lower with each additional copy of the *T* allele (model 1: HR 0.89; 95% CI, 0.81–0.98, p = 0.014; model 2: HR 0.85; 95% CI, 0.77–0.93, p = 4.6×10^−4^). Among 10,600 participants, 418 suffered a stroke (model 1: HR 0.94; 95% CI, 0.82–1.08, p = 0.363; model 2: HR 0.96; 95% CI, 0.83–1.11, p = 0.570) and 1,705 developed CHD (model 1: HR 0.97; 95% CI, 0.91–1.04, p = 0.422; model 2: HR 0.98; 95% CI, 0.91–1.05, p = 0.523) over a mean follow-up period of 15 years; rs780094 was not significantly associated with either outcome.

Among black study participants, 478 of 2,626 without diabetes at baseline developed diabetes over a mean follow-up period of 7 years. Figure 1B shows the cumulative incidence of diabetes stratified by rs780094 genotype: *TT* carriers had lower incidence of diabetes than *CC* carriers over the duration of follow-up, but the difference was not statistically significant. A stroke was suffered by 273 of 3,513 participants over a mean follow-up period of 15 years, and 408 participants developed CHD over this time period. rs780094 was not significantly associated with either outcome ([stroke: model 1: HR 0.97; 95% CI, 0.78–1.21, p = 0.79; model 2: HR 0.96; 95% CI, 0.77–1.120, p = 0.75], [CHD (model 1: HR 1.10; 95% CI, 0.93–1.31, p = 0.28; model 2: HR 1.08; 95% CI, 0.91–1.29, p = 0.37]).

A set of sensitivity analyses was conducted using a sub-sample of white participants without diabetes and who reported taking no lipid-lowering medications at baseline (Table S1). The associations of the rs780094 *T*-allele and most lipid and glycemic traits were strengthened. In contrast to analyses conducted on the overall sample, we observed an association of the *T* allele with higher systolic blood pressure (model 1: p = 0.016; model 2: p = 0.002). As multiple measurements of glucose, insulin, triglycerides, HDL, waist circumference and blood pressure are available in the ARIC Study, additional sensitivity analyses were conducted using mean values of these variables. Results remained similar with the exception of the association of the *T* allele with higher HDL cholesterol levels, which clearly became stronger (Table S2).

## Discussion

Using a large, population-based cohort, our study replicated all previous associations of the *GCKR* rs780094 polymorphism with metabolic traits, including fasting glucose, insulin, post-OGTT glucose, CRP, and triglyceride concentrations and diabetes prevalence among white participants. In addition, we observed associations of the rs780094 *T*-allele with decreased beta-cell function as assessed by HOMA-B and increased HDL cholesterol levels. The *T*-allele was nominally associated with increased prevalence of the metabolic syndrome. Among a large sample of African American participants, the variant was only marginally associated with serum triglyceride and insulin levels and HOMA-IR. Prospectively, among white participants the rs780094 *T*-allele was significantly associated with lower incidence of diabetes over a mean follow-up of 8 years, but not with incidence of CHD or stroke over a mean follow-up of 15 years. All associations were confirmed in sensitivity analyses using a subsample of participants without diabetes and not taking lipid-lowering medication. The observed associations with multiple metabolic traits therefore implicate the rs780094 variant as an example of pleiotropy emerging from GWAS, or may be indicative of a central connection between hepatic pathways of glucose and lipid metabolism.

This is one of the first and the largest study thus far investigating the association of rs780094 (or rs1260326, a variant in high linkage disequilibrium (LD)) and metabolic traits among African Americans[17]. As our findings did not extend to participants who self-identified as black, the intronic SNP rs780094 is likely not the causal variant itself but rather in LD with such a variant in European and Asian-ancestry populations but not African-ancestry populations. The *GCKR* LD block contains a non-synonymous coding SNP, rs1260326 (Pro446Leu). A previous fine-mapping effort within the region in European-ancestry individuals identified rs1260326 as the variant most strongly associated with triglyceride levels[17]. In addition, a recent functional study demonstrated that a Pro446Leu mutation in rs1260326 resulted in attenuated fructose-6-phosphate enhancement of GKRP activity in the liver [40]. rs1260326 is in strong LD with rs780094 in the HapMap CEU (r^2^ = 0.93) and CHB/JPT (r^2^ = 0.83) populations, but only moderate LD in the HapMap YRI sample (r^2^ = 0.43). In addition to differences in LD pattern, we also had less power to detect significant associations in the black study participants due to a smaller sample size and lesser frequency of the *T*-allele as compared to the white study participants. While the effect estimates for association with serum triglycerides were of comparable size among white and black study participants, the effect estimates for an association with most other traits were smaller among African Americans.

As in past studies, we did not observe an association of rs780094 with incident CHD and stroke despite the association of rs780094 with a number of cardiovascular risk factors, possibly because of the opposing effects of the SNP on the individual risk factors. The T-allele is simultaneously associated with lower fasting glucose, characteristic of a favorable CVD risk profile, and higher triglyceride and uric acid levels, characteristic of an unfavorable CVD risk profile. Alternatively, the small proportion of variance of the individual glycemic and lipid traits explained by rs780094 (less than 1% for all individual traits) may have contributed to the lack of association with incident cardiovascular outcomes we observed.

Similarly, rs780094 is simultaneously associated with both favorable and unfavorable components of the metabolic syndrome. The association of the *T*-allele with increased metabolic syndrome prevalence in white participants could be explained by the greater strength of the relationship with increased triglyceride levels as compared to lower glucose levels. This notion is supported by the fact that the association between the T allele and metabolic syndrome prevalence disappeared upon further adjustment for triglyceride concentrations.

GKRP is produced in molar excess over glucokinase in liver hepatocytes. Under conditions of low glucose, GKRP allosterically inhibits the phosphorylation of glucose to glucose-6-phosphate by glucokinase; the combined glucokinase/GKRP complex is subsequently sequestered in the nucleus [10]. Experimental observations in mice suggest this inhibition also functions to protect glucokinase against degradation by cytoplasmic proteases[41], [42]. The underlying mechanism of action of the *GCKR* polymorphism was proposed in a previous functional study, which observed that the Pro446Leu mutation at rs1260326 resulted in attenuation of fructose-6-phosphate enhancement of GKRP activity[43]. Under this model, lower fasting glucose levels could occur as a result of increased glycolytic flux due to lowered inhibition of glucokinase by GKRP. Simultaneously, the body's ability to respond to a postprandial glucose surge is weakened because of a smaller pool of reserve glucokinase. These expectations are in line with the rs780094 T-allele's opposing and simultaneous associations with lower fasting and higher post-OGTT glucose levels. In addition, we observed a trend towards an association of the *T*-allele with decreased beta-cell function as assessed by HOMA-B, which became more significant after further adjusting for triglyceride levels. This association has not been observed in most other studies[13], [18], and may suggest an as-of-yet unknown function of GKRP in pancreatic beta cells, where *GCKR* is expressed at low levels in comparison to *GCK* [40]. Another study in Han Chinese found a similar association in the opposite direction[25]. In that study, the minor allele of rs780094 was associated with increased insulin levels, in contrast to the findings of most studies of *GCKR* thus far[12], [16], [18].

The strengths of our study include the large size of the ARIC Study, the inclusion of both white and African-American individuals, the availability of a wide range of metabolic risk factors, and the inclusion of prospective data on incident diabetes, CHD and stroke. We also note the limitations of our study. First, we examined only one SNP, rs780094, in the *GCKR* gene. However, rs780094 has been replicated consistently and is in high LD with a reportedly functional variant, rs1260326, in European-ancestry populations. We chose a conventional significance threshold of 0.05 to evaluate the association between one exposure and several pre-specified outcomes; correction for multiple testing would not have changed the conclusions for the highly significant associations we observed. Second, baseline measurements for OGTT and CRP were unavailable and we therefore used data collected an average of 9 years later. However, there is no evidence of a differential effect of rs780094 with respect to age, and covariates were always taken from the same visit at which an outcome was measured.

In conclusion, our study provides evidence of a common *GCKR* variant associated with multiple metabolic traits including glycemic and lipid traits and CRP levels in white ARIC Study participants. Among black study participants, only the associations with triglycerides, insulin and HOMA-IR were nominally significant, suggesting that rs780094 of *GCKR* is not a causal variant. The rs780094 *T*-allele was associated with lower diabetes prevalence and incidence; it was not associated with incident CHD or stroke.

## Supporting Information

Table S1 Adjusted Associations between rs780094 and Study Outcomes in 9,356 White Atherosclerosis Risk in Communities Study Participants Without Diabetes and Not Taking Lipid-Lowering Medications at Baseline. * Model 1 adjusted for age, sex, study center; model 2 adjusted for age, sex, study center, fasting glucose, fasting insulin, triglycerides. HOMA-IR, HOMA-B, and DM not adjusted for fasting glucose and insulin in model 2. All cross-sectional associations evaluated using baseline measurements (n = 9,356), except for 2 hr post-OGTT glucose (n = 5,674) and C-reactive protein (n = 6,086), which were only measured at visit 4. 890 participants out of 8,917 developed DM over mean follow-up of 8 years; 1,291 out of 9,356 developed CHD and 319 out of 9,356 experienced a stroke over mean follow-up of 15 years.
(0.04 MB DOC)

Table S2 Effect of using repeated measurements versus cross-sectional measurements to evaluate the association between rs780094 and different outcomes in 10,601 White Atherosclerosis Risk in Communities Study Participants. Model 2 adjusted for age, sex, study center, insulin, fasting glucose, triglycerides. The right-hand side of the table presents results for mean values of insulin, glucose, and triglycerides, and adjusting for mean levels of covariates. Repeated measurements were available from ARIC visits 1 and 4 for insulin, from ARIC visits 1, 2 and 4 for glucose, and for ARIC visits 1, 2, 3, and 4 for triglycerides, waist circumference, HDL cholesterol, and systolic blood pressure. *not adjusted for fasting glucose and insulin.
(0.04 MB DOC)
