# Supplementary material for: Association of rs780094 in GCKR with Metabolic Traits and Incident Diabetes and Cardiovascular Disease: The ARIC Study
Source: PLoS One. 2010 Jul 22;5(7):e11690. doi: 10.1371/journal.pone.0011690 (PMC2908550; doi:10.1371/journal.pone.0011690)
Supplement: Table S1 — Adjusted Associations between rs780094 and Study Outcomes in 9,356 White Atherosclerosis Risk in Communities Study Participants Without Diabetes and Not Taking Lipid-Lowering Medications at Baseline. * Model 1 adjusted for age, sex, study center; model 2 adjusted for age, sex, study center, fasting glucose, fasting insulin, triglycerides. HOMA-IR, HOMA-B, and DM not adjusted for fasting glucose and insulin in model 2. All cross-sectional associations evaluated using baseline measurements (n = 9,356), except for 2 hr post-OGTT glucose (n = 5,674) and C-reactive protein (n = 6,086), which were only measured at visit 4. 890 participants out of 8,917 developed DM over mean follow-up of 8 years; 1,291 out of 9,356 developed CHD and 319 out of 9,356 experienced a stroke over mean follow-up of 15 years. (0.04 MB DOC) [file pone.0011690.s001.doc]

**Table S1: Adjusted Associations between rs780094 and Study Outcomes in 9,356 White Atherosclerosis Risk in Communities Study Participants Without Diabetes and Not Taking Lipid-Lowering Medications at Baseline**

|  | **Model 1*** | | **Model 2*** | |
| --- | --- | --- | --- | --- |
| **Quantitative traits** | **Effect/T allele ± SE** | **P** | **Effect/T allele ± SE** | **P** |
| Triglycerides (mmol/l) | +0.12 ± 0.01 | 3.0x10^-22^ | +0.15 ± 0.01 | 2.2x10^-35^ |
| Fasting glucose (mg/dl) | -0.71 ± 0.1 | 3.7x10^-8^ | -0.59 ± 0.1 | 1.8x10^-6^ |
| Fasting insulin (pmol/l) | -3.93 ± 0.8 | 5.9x10^-7^ | -4.96 ± 0.7 | 2.7x10^-12^ |
| HOMA-IR | -0.17 ± 0.03 | 3.6x10^-8^ | -0.27 ± 0.03 | 1.2x10^-20^ |
| HOMA-B | -2.73 ± 1.1 | 0.009 | -5.96 ± 1.0 | 2.5x10^-9^ |
| Waist circumference (cm) | +0.05 ± 0.2 | 0.777 | +0.38 ± 0.2 | 0.018 |
| HDL cholesterol (mg/dl) | -0.17 ± 0.2 | 0.451 | +0.35 ± 0.2 | 0.084 |
| Systolic blood pressure (mmHg) | +0.57 ± 0.2 | 0.016 | +0.72 ± 0.2 | 0.002 |
| C-reactive protein (mg/l) | +0.55 ± 0.1 | 1.1x10^-6^ | +0.55 ± 0.1 | 1.1x10^-6^ |
| 2 hr post-OGTT glucose (mg/dl) | +3.06 ± 0.8 | 4.9x10^-5^ | +2.87 ± 0.7 | 3.6x10^-5^ |
| **Incident events** | **HR/T allele (95% CI)** | **P** | **HR/T allele (95% CI)** | **P** |
| Diabetes mellitus | 0.88 (0.80-0.97) | 0.009 | 0.84 (0.76-0.93) | 3.9x10^-4^ |
| Coronary heart disease | 0.97 (0.90-1.05) | 0.454 | 0.96 (0.88-1.04) | 0.270 |
| Stroke | 0.97 (0.83-1.14) | 0.730 | 0.99 (0.84-1.16) | 0.858 |
