# Supplementary material for: Association of rs780094 in GCKR with Metabolic Traits and Incident Diabetes and Cardiovascular Disease: The ARIC Study
Source: PLoS One. 2010 Jul 22;5(7):e11690. doi: 10.1371/journal.pone.0011690 (PMC2908550; doi:10.1371/journal.pone.0011690)
Supplement: Table S2 — Effect of using repeated measurements versus cross-sectional measurements to evaluate the association between rs780094 and different outcomes in 10,601 White Atherosclerosis Risk in Communities Study Participants. Model 2 adjusted for age, sex, study center, insulin, fasting glucose, triglycerides. The right-hand side of the table presents results for mean values of insulin, glucose, and triglycerides, and adjusting for mean levels of covariates. Repeated measurements were available from ARIC visits 1 and 4 for insulin, from ARIC visits 1, 2 and 4 for glucose, and for ARIC visits 1, 2, 3, and 4 for triglycerides, waist circumference, HDL cholesterol, and systolic blood pressure. *not adjusted for fasting glucose and insulin. (0.04 MB DOC) [file pone.0011690.s002.doc]

**Table S2: Effect of using repeated measurements versus cross-sectional measurements to evaluate the association between rs780094 and different outcomes in 10,601 White Atherosclerosis Risk in Communities Study Participants**

|  | **Visit 1 cross-sectional measures, model 2** | | **Mean of repeated measures, model 2** | |
| --- | --- | --- | --- | --- |
| **T** | **Effect/*T* allele ± SE** | **P** | **Effect/*T* allele ± SE** | **P** |
| Triglycerides (mmol/l) | +0.16 ± 0.01 | 2.4x10^-31^ | +0.17 ± 0.01 | 2.2x10^-46^ |
| Fasting glucose (mg/dl) | -1.93 ± 0.4 | 2.3x10^-7^ | -2.13 ± 0.4 | 5.5x10^-9^ |
| Fasting insulin (pmol/l) | -6.29 ± 1.3 | 1.9x10^-6^ | -6.83 ± 1.3 | 5.2x10^-8^ |
| HOMA-IR* | -0.45 ± 0.1 | 2.2x10^-9^ | -0.48 ± 0.1 | 9.6x10^-12^ |
| HOMA-B* | -8.36 ± 3.3 | 0.011 | -7.86 ± 3.3 | 0.016 |
| Waist circumference (cm) | +0.01 ± 0.2 | 0.940 | -0.02 ± 0.2 | 0.903 |
| HDL cholesterol (mg/dl) | +0.43 ± 0.2 | 0.022 | +0.78 ± 0.2 | 6.4x10^-6^ |
| Systolic blood pressure (mmHg) | +0.30 ± 0.2 | 0.188 | +0.32 ± 0.2 | 0.127 |
